# The role of WWOX polymorphisms on COPD susceptibility and pulmonary function traits in Chinese: a case-control study and family-based analysis

**DOI:** 10.1038/srep21716

**Published:** 2016-02-23

**Authors:** Chenli Xie, Xiaoliang Chen, Fuman Qiu, Lisha Zhang, Di Wu, Jiansong Chen, Lei Yang, Jiachun Lu

**Affiliations:** 1 The State Key Lab of Respiratory Disease, The Institute for Chemical Carcinogenesis, Collaborative Innovation Center for Environmental Toxicity, Guangzhou Medical University, 195 Dongfengxi Road, Guangzhou 510182, China; 2 Department of respiratory medicine, The Fifth People’s Hospital of Dongguan City, Dongguan 523900, China; 3 Shenzhen Guangming district center for disease control and prevention Shenzhen 518106, China

## Abstract

Single nucleotide polymorphisms (SNPs) in the WW domain containing oxidoreductase (*WWOX*) gene were recently identified to be quantitative trait loci for lung function and thus likely to be susceptible biomarkers for COPD. However, the associations between *WWOX* SNPs and COPD risk are still unclear. Here, by conducting a two-center case-control study including 1511 COPD cases and 1677 controls and a family-based analysis comprising 95 nuclear pedigrees, we tested the associations between five SNPs that are rs10220974C >T, rs3764340C >G, rs12918952G >A, rs383362G >T, rs12828G >A of *WWOX* and COPD risk as well as the hereditary inclination of these loci among COPD families. We found that the SNP rs383362G >T was significantly associated with an increased risk of COPD in a T allele-number dependent-manner (OR = 1.30, 95%CI = 1.11 - 1.52). The T allele was more prone to over transmit to sick children and sibs than the G allele (Z = 2.900, *P* = 0.004). Moreover, the forced expiratory volume in one second*/*forced vital capacity (FEV1/FVC), FEV1/predicted-FEV1 and annual FEV1 also significantly decreased in the rs383362T carriers compared to the rs383362GG carriers. For other SNPs, no significant association was observed for COPD and pulmonary function. Taken together, our data demonstrated that the SNP rs383362G >T of *WWOX* plays a role in COPD inheritance.

Chronic obstructive pulmonary disease (COPD) is one of the most strikingly increasing lung diseases characterized by incompletely reversible airflow obstruction. During 1990–2010, COPD ranked the forth and the third causes of death in American and in Chinese, respectively^1^,^2^. The values are still ongoing now and no doubt that COPD will seriously influence the life quality of and life health of patients and cause high economic burden on patients as well as the society.

The pathogenesis of COPD is complex involving both environmental and genetic factors. Environmental factors such as tobacco smoking and air pollution can cause a series of sophisticated biological reactions like oxidative stress to induce COPD development, while genetic variants can regulate the expression or function of such molecules participating in above reactions and thus determinate COPD susceptibility^3^. Tobacco smoking can bring a large amount of oxygen free radical into lung and trigger oxidative stress, which directly damages the lung tissue to participate in the pathological courses of COPD^4^,^5^,^6^. In response to the damage, the organism activates the antioxidant system to resist the harm of oxygen free radical such as up-expression of a series of antioxidant enzymes in airway epithelial cells^7^,^8^,^9^. Due to different genetic background, different smokers show significantly different expression or function of these enzymes^10^,^11^,^12^. In this condition, genetic variants located in these antioxidant enzymes might alter individuals’ susceptibility to develop COPD^12^,^13^,^14^.

Recent results from the genome-wide association studies (GWASs) verified above opinion. Two GWASs conducted in European reported that single nucleotide polymorphisms (SNPs), which are located in the WW domain containing oxidoreductase (*WWOX*) gene, were significantly associated with pulmonary function traits including the forced expiratory volume in one second (FEV1), forced vital capacity (FVC) and FEV1/FVC^15^,^16^. WWOX is a potential antioxidant enzyme with respect to its oxidoreductase functional domain. WWOX plays important roles in the regulation of a wide variety of cellular functions such as protein degradation, transcription and RNA splicing. Stimulus such as tobacco smoking can directly trigger oxidative stress by virtue of decrease in WWOX expression^7^. WWOX also can interact with P53 that is an important regulator involving the pathogenesis of COPD^17^. Taken together, these evidences suggest the *WWOX* gene to be a susceptible gene of COPD.

However, association between *WWOX* SNPs and COPD risk are still unknown by now. Also, the GWASs reported SNPs are located in the introns of *WWOX*, which are lack of possibly biological function. The human *WWOX* gene (NM_016373) maps to the chromosome 16q23.3–24.1 region with a 1.13Mb DNA sequence. Through the fine mapping analysis, we had previously reported that the *WWOX* SNPs were associated with lung cancer risk in Chinese^18^. Overlap of susceptible loci between COPD and lung cancer had been widely observed^13^,^18^,^19^,^20^. Therefore, we aimed to test the hypothesis that *WWOX* SNPs were associated with COPD risk in Chinese.

In the current study, we conducted a two-stage case-control study including 1511 COPD cases and 1677 controls to test the associations between five putatively functional tagSNPs of *WWOX* (i.e., rs10220974C >T, rs3764340C >G, rs12918952G >A, rs383362G >T and rs12828G >A) and COPD risk. We further performed a family-based analysis comprising 95 pedigrees to verify the associations and to assess the hereditary inclination of susceptible loci among COPD pedigrees.

## Results

### Associations between the WWOX SNPs and COPD risk

Supplementary Table S1 summarizes frequency distribution of demographic characteristics, possible risk factors and the Global Initiative for Chronic Obstructive Lung Disease (GOLD) stages in COPD cases and controls of both populations.

Table 1 presents genotypes of the five *WWOX* SNPs between cases and controls in the southern Chinese population as a discovery set. All genotype frequency distributions of these SNPs matched the Hardy-Weinberg equilibrium (HWE) in the controls (*P* > 0.05 for all). As shown, only a significant deviation was found in genotype frequency distribution of the SNP rs383362G >T between the COPD cases and controls (*P* = 0.025), but was not of others (*P* > 0.05 for all). Compared to the rs383362GG genotype, the rs383362TG genotype exerted a significantly increased COPD risk (OR = 1.25, 95%CI = 1.01–1.55), while the the rs383362TT genotype conferred a non-significant increased COPD risk (OR = 2.23, 95%CI = 0.84–5.93). According to the genetic model selection strategy^21^, this SNP best fitted the additive model that the rs383362G >T conferred a significantly increased risk to COPD in a T allele dose-dependent manner (OR = 1.28, 95%CI = 1.06–1.56). However, the Bonferroni correction test did not support the significance of the association (*P* = 0.125). In addition, because one previous study had reported the haplotype rs3764340G-rs383362T with significant association with lung cancer risk^18^, we tested the association between the haplotype and COPD risk. However, no significant association was observed (Data not shown).

Despite the Bonferroni correction showed no significant association between the SNP rs383362G >T and COPD risk, the above data still supported a promising association between them considering that the Bonferroni correction sometimes cause excessive correction. Thus, we still validated the SNP in the eastern Chinese population. The results were consistent. The SNP was associated with an increased COPD risk under the additive model (OR = 1.36, 95%CI = 1.04–1.77; Bonferroni correction: *P* = 0.130; Table 2). We then merged the two populations to increase the study power. Under the additive model, the risk for developing COPD increased by 1.30-times accompanying with the number of rs383362T allele (OR = 1.30, 95%CI = 1.11–1.52; Bonferroni correction: *P* = 0.005; Table 2).

Table 3 shows results from stratification analysis of the association between the SNP rs383362G > T and COPD risk, as well as the interaction between the SNP and variables on COPD risk. The rs383362G > T significantly interacted with biomass as fuels on increasing COPD risk (*P* = 0.021), because there was an intuitionistic higher OR that equaled to 2.32 (95%CI = 1.28–4.22) in subjects who used biomass as fuels than the OR that equaled to 1.23 (95%CI = 1.04–1.45) in subjects who did not use biomass as fuels. Further three-way interaction analysis showed no significant interaction between biomass as fuels, gender and the SNP on COPD risk (*P* = 0.548). No other significant interaction was observed (*P* > 0.05 for all). We also conducted a multivariable logistic regression analysis including above covariates and interaction. The data showed a 1.31-fold risk of COPD in rs383362T carriers compared to rs383362GG carriers (OR = 1.31, 95%CI = 1.12–1.55).

### Heritability inclination of WWOX SNPs among COPD families

As shown in Table 4, the single-marker analysis indicated that the T risk allele of rs383362G > T was more prone to over transmitted to sick children and sibs under the additive genetic model (Z = 2.900, *P* = 0.004), so other variants were not (*P* > 0.05 for all). The haplotype analysis involving the two SNPs rs3764340C >G and rs383362G >T further demonstrated that compared to the other three haplotypes, the T-C haplotype exerted significantly excessive transmission from parents to affected offspring under the additive genetic model (Z = 2.550, *P* = 0.011), so other haplotypes did not (*P* > 0.05 for all). In addition, the logistic regression model showed that the rs383362T allele and T-C haplotype conferred significantly increased risk of COPD compared to the rs383362G allele (OR = 2.04, 95%CI = 1.41–2.95) and G-C haplotype (OR = 1.64, 95%CI = 1.11–2.41), respectively.

### Effect of WWOX SNPs on pulmonary function traits

**Figure 1:**
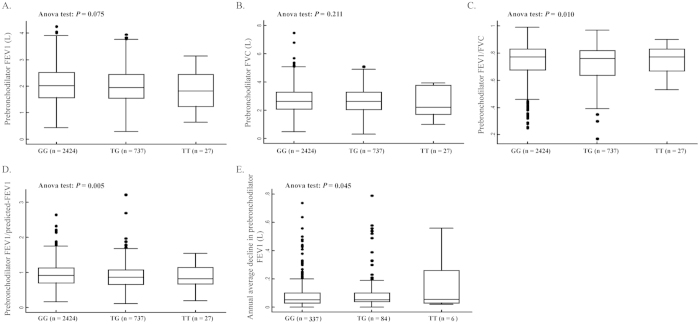
Effect of the *WWOX* SNP rs383362G >T on pulmonary function traits. (**A**) Effect on prebronchodilator FEV1; (**B**) Effect on prebronchodilator FVC; (**C**) Effect on prebronchodilator FEV1/FVC; **(D**) Effect on prebronchodilator FEV1/predicted-FEV1; (**E**) Effect on annual average decline of prebronchodilator FEV1. Columns, mean from individuals’ lung function; FEV1: forced expiratory volume in 1 second; FVC: forced vital capacity. Bars, SD; The oneway ANOVA test was used to test the differences in pulmonary function between the rs383362G >T genotype carriers. As shown, the rs383362T variants significantly exerted lower FEV1 and FEV1/predicted-FEV1, and caused more annual average decline in FEV1 than the rs383362GG genotype.

We examined effect of the five *WWOX* SNPs on prebronchodilator pulmonary function traits including FEV1, FVC, FEV1/FVC and FEV1/predicted-FEV1 in the total of 3188 studied subjects and annual average decline of FEV1 in the 427 individuals with follow-up of pulmonary function. Apparently, decreases in almost all lung function traits were observed along with number of the rs383362T allele, as that was the mean FEV1 (GG, TG, TT: 2.032 ± 0.683, 1.981 ± 0.712, 1.830 ± 0.773), mean FVC (GG, TG, TT: 2.714 ± 0.851, 2.676 ± 0.867, 2.472 ± 1.051), mean FEV1/FVC (GG, TG, TT: 0.744 ± 0.125, 0.728 ± 0.131, 0.753 ± 0.111) and mean FEV1/predicted-FEV1 (GG, TG, TT: 0.920 ± 0.332, 0.876 ± 0.355, 0.855 ± 0.370) that became progressively smaller from the GG carriers, TG carriers to TT carriers. However, the trend was only statistically significant in FEV1/FVC (ANOVA test: *P* = 0.010) and FEV1/predicted-FEV1 (*P* = 0.005), but not in FEV1 (*P* = 0.075) and FVC (*P* = 0.211). Moreover, the rs383362T variants significantly caused more annual average decline in FEV1 than the rs383362GG genotype (TT vs. TG vs. GG: 0.163 ± 0.214, 0.113 ± 0.151, 0.086 ± 0.101, *P* = 0.045; Fig. 1E). In addition, non-significant correlation was observed between the other SNPs and each lung function trait (Data not shown).

### Possible function of the rs383362G >T polymorphism by bioinformatics analysis

The SNP rs383362G >T is located in 3′-untranslated region (3′-UTR) of *WWOX* that may affect binding ability of potentially microRNA or RNA-binding protein and thus regulates WWOX expression. By querying the SNPexp database (http://app3.titan.uio.no/biotools/tool.php?app=snpexp) with regard to the CHB population (i.e., a Chinese population), the mean WWOX expression was consistently lower in 8 cases of lymphoblastoid cells with the rs383362TG genotype than that in 37 cases with the rs383362GG genotype using different expression microarray platforms. However, the difference was not significant, which may be due to the limited sample size. Also, by polling the SNPinfo website (http://snpinfo.niehs.nih.gov/snpfunc.html), the transversion of G to T may influence four microRNAs’ binding ability to WWOX 3′-UTR. Interestingly, two microRNAs among the four microRNAs, hsa-miR-134 and hsa-miR-758 were experimentally proved to be translational regulators of WWOX (http://www.genecards.org/cgi-bin/carddisp.pl?gene=WWOX).

## Discussion

In the current study, based on a two-stage case-control and a family-based study, we found that the T genotypes of *WWOX* SNP rs383362G >T were significantly associated with an increased risk of COPD and decreased pulmonary function traits in Chinese. The T allele exerted significantly excessive transmission from parents to sick offspring. The genotypes also interacted with biomass as fuels on increasing COPD risk. Bioinformatics analysis further showed that the SNP has a possible regulation on WWOX expression.

COPD is a well-established disease involving both genetic and environmental factors. Multiple association studies especially the GWASs, have reported an abundant of SNPs to be susceptible loci for COPD or pulmonary function. These SNPs majorly belong to genes that involve the major pathogenic mechanisms of COPD, such as inflammation^22^,^23^, oxidative stress^15^,^24^, DNA damage^25^,^26^ and epithelial-mesenchymal transition (EMT)^26^,^27^. However, most of these SNPs are considered to be lack of function and non-causal variant with regard to their genomic location in gene introns or intergenic region. An effective strategy to discover causal variants was fine-mapping based on the results from GWASs. Fine mapping majorly analyzes those putatively function variants considering their location in cis-acting element of genes that harbor susceptible loci of disease. Based on this strategy, we found a 3′-UTR SNP rs383362G >T of *WWOX*, a gene that has been reported to harbor susceptible loci of pulmonary function by GWASs^15^,^16^, was associated with COPD risk and pulmonary function traits. The rs383362T risk allele was more prone to over transmitted to sick children and sibs. This SNP got Bioinformatics data support to have potentiality on affecting WWOX expression. Taken together, all these evidences suggested the rs383362G >T polymorphism to be a susceptible loci for COPD in Chinese.

Low expression of WWOX has been reported to be an inducement of human diseases such as lung cancer^28^,^29^,^30^. The SNP rs383362G >T is located in the 3′-UTR of *WWOX*, the transversion of G to T might influence four microRNAs’ binding ability to WWOX mRNA as bioinformatics analysis shown. Of the four SNPs, the hsa-miR-134 is an identified microRNA to regulate WWOX expression^31^. Meaningfully, expression data in lymphoblastic cells of CHB from microarray platforms showed that the rs383362T variants have lower WWOX expression than the rs383362GG genotype. These indicated a conjectural mechanism underlying the effect of rs383362G >T to COPD susceptibility. The SNP also interacted with biomass as fuels. Biomass exposure is an important risk factor of COPD, from which smoke can produce environmental stimuluses and induce oxidative damage^32^. WWOX can be downregulated by such exposures^33^. Therefore, subjects with T genotypes are more vulnerable to suffer oxidative damage and thus predispose to develop COPD.

Associations between the *WWOX* SNPs and several human diseases have been reported including thyroid carcinoma^34^, prostate cancer^35^,^36^, breast cancer^37^, and lung cancer^18^. Now, we identified the *WWOX* SNP rs383362G >T to be associated with COPD risk. Meanwhile, the adverse effect driven by the rs383362T allele was increased by a slight in patients with GOLD stage III than those with GOLD stage II or I. The effect was not significant in stage IV, which may be due to the limited sample size. Furthermore, the SNP rs383362G >T was also correlated with FEV1/FVC and FEV1/predicted-FEV1, both of which are essential on COPD diagnosis. The rs383362T variants also caused more annual average decline in FEV1 than the rs383362GG genotype. Decline in FEV1 is a marker for airflow obstruction and serves as primary symptom of both developing COPD and exacerbation. These correlations suggested that the SNP has direct effect on pulmonary function, further supporting its association with COPD risk. Taken together, The SNPs might be not only a genetic biomarker for COPD onset but also a predictor for COPD severity.

Some limitations may affect the validity of current study. First, the portion of GOLD stage in our study was a little inconsistent with the parent population as suggested with respect to the high frequency of stage I COPD in the current study, suggesting a serious selection bias in the current study that influenced the external validity^38^. Second, some important confounders such as passive smoking were not concerned in this study, which might cause confounding bias and thus affect internal validity. Third, the relatively small sample size of the nuclear pedigrees in family based analysis might also cause error results. However, the family-based analysis effectively controlled some confounding biases. Because consistent associations were observed in both the case-control study and family based analysis, and the SNP got Bioinformatics data support to be functional, it is conceivable that our finding was not achieved by chance.

To conclude, our study identified a putatively functional SNP rs383362G >T of *WWOX* that confers an increased risk of COPD, the rs383362T risk allele was more prone to over transmitted from parents to sick offspring. This SNP might be a genetic biomarker to predict risk of COPD in Chinese.

## Materials and Methods

### Case-control study

A two-stage case-control study was conducted in a southern Chinese population and an eastern Chinese population, which have been described in previously published studies^13^,^14^. COPD patient was defined as subjects with FEV1/FVC <70% after inhalation of 400μg salbutamol and with some chronic airway symptoms including chronic cough, dyspnea, sputum production or wheezing. In brief, the southern Chinese population included 1025 COPD patients and 1061 age (±5 years) and sex frequency-matched controls, of which 697 cases were recruited from three communities (Liwan, Xicun and Zhanqian communities) in Guangzhou city based on annual cross-sectional surveys of COPD, 328 cases were recruited from physical examination centers of three hospitals in Guangzhou city including the Guangzhou Chest Hospital, the third Affiliated Hospital of Guangzhou Medical University and the third Affiliated Hospital of Sun Yat-sen University. All controls were selected from individuals with normal lung function (i.e., FEV1/FVC >70%) who participated in the annual cross-sectional surveys of COPD. The eastern Chinese population comprised 486 COPD patients and 616 normal controls, of which the cases were enrolled from the physical examination center of the Second Affiliate Hospital of Soochow University and the controls were selected from a database consisting of 3,500 individuals based on a physical examination in Suzhou city. All subjects are generally unrelated Han Chinese. Moreover, a long-term follow-up on lung function monitoring was performed in part of the southern Chinese population for years as previously reported^39^. Heretofore, 427 individuals were successfully followed up and had at least four years’ data of lung function between 2002 and 2010 with annual spirometry test. Each subject was scheduled for an interview with a structured questionnaire to provide data on age, sex, smoking status, pack-years smoked, biomass using and to donate 5 mL peripheral blood after a written informed consent had obtained. The definitions of smoking status and biomass as fuels were described in our previous publications^13^. Briefly, those participants who had smoked <100 cigarettes in their lifetime were defined as never smokers. Otherwise, they were classified as ever smokers. Biomass as fuels means that people use bio-crop stalks and wood as fuels. The study was approved by the institutional review boards of Guangzhou Medical University and Soochow University. The methods were carried out in accordance with the approved guidelines.

### Family-based analysis

A family-based analysis was conducted in a southern Chinese population between September 2010 and September 2012. COPD diagnosis was consistent with the above COPD definition. Briefly, 148 COPD probands were originally recruited from the Fifth People’s Hospital of Dongguan City. Among them, 113 were males with a mean age as 67.9 ± 12.9 year-old and 35 were females with a mean age as 65.6 ± 16.3 year-old. The probands’ immediate family members who were mostly the probands’ brothers, sisters and offspring were then asked to take a diagnostic test of COPD. To the exclusion of those who did not finish or achieve lung function test, 342 individuals were enrolled included 69 COPD cases and 273 healthy people. According to the definition of nuclear family, there were 95 nuclear families in the total population (n = 308), of which 26 (27.4%, n = 88) families had both parents, 29 (30.5%, n = 95) families had one single parent and at least one brother or sister, and 40 (42.1%, n = 125) had no available parents but at least two brothers or sisters. For these 95 COPD probands of nuclear families, 70 were males with a mean age as 67.0 ± 12.5 year-old and 25 were females with a mean age as 65.8 ± 17.9 year-old. All subjects are generally unrelated Han Chinese. Each subject provided data using a questionnaire as above described and donated 5 mL peripheral blood after a written informed consent was obtained. The analysis was approved by the institutional review boards of Guangzhou Medical University. The methods were carried out in accordance with the approved guidelines.

### SNPs selection and genotyping

We selected 5 putatively functional tagSNPs that are rs10220974C >T in promoter, rs3764340C >G, rs12918952G >A in exons, rs383362G >T, rs12828G >A in 3′-UTR as previously described^18^. The 5 SNPs were genotyped using the TaqMan allelic discrimination Assay on the ABI7500 system with self-designed primes and probes as shown in Supplementary Table S2 18.

### Statistical analysis

The chi-square test was used to assess differences in the frequency distribution of SNPs’ genotypes between the cases and controls. The PROC HAPLOTYPE procedure in SAS/Genetics software was used to infer haplotype frequencies from the observed genotypes of SNPs. The unconditional logistic regression model without or with adjustment for these covariates, which were significantly associated with COPD risk including age, sex, pack-years smoked, biomass as fuels and sample source region, was used to estimate the odds ratio (OR) and 95% confidence interval (95%CI) to measure effect of each SNP as well as haplotype on COPD risk. The multiplicative interaction analysis was applied for assessing the interaction between each SNP and surrounding factors^40^. The family base-association test (FBAT) software was used to perform transmission disequilibrium test (TDT) and transmission disequilibrium of patient-normal (SDT) to analysis the effect of *WWOX* SNPs and haplotypes on susceptibility of COPD^41^. The oneway ANOVA test was performed to assess the effect of *WWOX* SNPs on pre-bronchodilator pulmonary function traits. All tests were two-sided by using the SAS software (version 9.2; SAS Institute, Cary, NC). *P* < 0.05 was considered to be statistically significant.

## Additional Information

**How to cite this article**: Xie, C. *et al.* The role of WWOX polymorphisms on COPD susceptibility and pulmonary function traits in Chinese: a case-control study and family-based analysis. *Sci. Rep.* **6**, 21716; doi: 10.1038/srep21716 (2016).

## Supplementary Material

Supplementary Table S1-2

## Figures and Tables

**Table 1:** Genotype distributions of *WWOX* SNPs and their associations with COPD risk in the southern Chinese population.

SNP	Location in gene	Case^a^ (n = 1025)	Control^a^,^b^ (n = 1061)	*P*^c^	MAF^d^		OR _*het*_^e^ (95%CI)	OR _*hom*_^e^ (95%CI)	OR _*add*_^e^ (95%CI)	OR _*dom*_^e^ (95%CI)	OR _*rec*_^e^ (95%CI)
					Case	Control					
rs10220974C >T	Promoter	748/252/25	806/231/24	0.290	0.147	0.132	1.20 (0.97–1.48)	1.02 (0.57–1.83)	1.13 (0.95–1.35)	1.18 (0.96–1.45)	0.98 (0.55–1.75)
rs3764340C >G	Exon	832/186/7	837/214/10	0.390	0.098	0.110	0.92 (0.73–1.15)	0.71 (0.26–1.94)	0.91 (0.74–1.12)	0.91 (0.73–1.13)	0.73 (0.27–1.97)
rs12918952G >A	Exon	884/137/4	910/144/7	0.688	0.071	0.075	1.01 (0.78–1.31)	0.66 (0.19–2.29)	0.98 (0.77–1.25)	1.00 (0.77–1.29)	0.66 (0.19–2.28)
rs383362G >T	3′-UTR	755/259/11	834/220/7	0.025	0.137	0.110	1.25 (1.01–1.55)	2.23 (0.84–5.93)	1.28 (1.06–1.56)	1.28 (1.04–1.57)	2.13 (0.80–5.64)
rs12828G >A	3′-UTR	474/463/88	473/489/99	0.692	0.312	0.324	0.94 (0.78–1.13)	0.95 (0.69–1.31)	0.96 (0.84–1.10)	0.94 (0.79–1.12)	0.98 (0.72–1.34)

Abbreviation: MAF, minor allele frequency; OR _*het*_, heterozygote versus wild-genotype homozygote; OR _*hom*_, variant homozygote versus wild-type homozygote; OR_*add*_, OR _*dom*_, OR _*rec*_ calculated by the additive, dominant and recessive model, respectively; 3′-UTR, 3′-untranslated region.

^a^Wild-type homozygote/heterozygote/variant homozygote.

^b^The observed genotype frequencies among the controls all matched the Hardy-Weinberg equilibrium in the control subjects (*P* > 0.05 for all).

^c^*P* values of a two-sided χ^2^ test for genotypes distribution between the cases and controls.

^d^MAF of the variant allele.

^e^Data were calculated by the unconditional logistic regression, adjusted for age, sex, pack-years smoked, biomass as fuels and sample source region.

**Table 2:** Association between the SNP rs383362G >T and COPD risk.

Genotype/Allele	Case n (%)	Control n (%)	Crude OR (95%CI)	Adjusted OR (95%CI)^b^
Eastern Chinese (Validation set)				
Total No.	486	616		
rs383362G >T				
GG	354(72.8)	481(78.1)	1.00 (ref.)	1.00 (ref.)
TG	126(16.0)	132(21.4)	1.30(0.98–1.72)	1.32(0.99–1.75)
TT	6(1.2)	3(0.5)	2.72(0.68–10.9)	2.77(0.67–11.5)
*P*^a^			0.075	
T allele	0.142	0.112		
Additive model				
TT vs. TG vs. GG			1.34(1.03–1.74)	1.36(1.04–1.77)
Merged population				
Total No.	1511	1677		
rs383362G >T				
GG	1109(73.4)	1315(78.4)	1.00 (ref.)	1.00 (ref.)
TG	385(25.5)	352(21.0)	1.30(1.10–1.53)	1.26(1.07–1.50)
TT	17(1.1)	10(0.6)	2.02(0.92–4.42)	2.39(1.07–5.35)
*P*^a^			0.002	
T allele	0.138	0.111		
Additive model				
TT vs. TG vs. GG			1.31(1.13–1.53)	1.30(1.11–1.52)

^a^*P* values of a two-sided χ^2^ test for genotypes distribution between the cases and controls.

^b^Adjusted in a logistic regression model that included age, sex, pack-years smoked, biomass as fuels and sample source region.

**Table 3:** Stratification analysis of the association between the SNP rs383362G >T and COPD risk in the merged population.

Factors	Case (*n* = 1511)			Control (*n* = 1677)			Adjusted OR (95% CI)^a^	*P*^b^
	TT	TG	GG	TT	TG	GG	TT vs. TG vs. GG	
Age (Years)								
≤60	6	186	512	5	176	618	1.26(0.99–1.59)	0.121
>60	11	199	597	5	176	697	1.34(1.07–1.68)	
Sex								
Male	11	230	642	4	216	763	1.30(1.06–1.61)	0.991
Female	6	155	467	6	136	552	1.28(1.00–1.65)	
Smoking status								
Never	10	203	584	8	212	806	1.32(1.07–1.62)	0.825
Ever	7	182	525	2	140	509	1.28(1.00–1.65)	
Pack-years smoked								
≥20	5	133	359	2	78	303	1.47(1.07–2.02)	0.866
<20	2	49	166	0	62	206	1.00(0.65–1.53)	
0	10	203	584	8	212	806	1.32(1.07–1.63)	
Biomass as fuels								
No	16	310	978	10	331	1226	1.23(1.04–1.45)	0.021
Yes	1	75	131	0	21	89	2.32(1.28–4.22)	
GOLD Stages								
I	5	158	458	10	352	1315	1.25(1.01–1.54)	
II	6	151	437				1.29(1.05–1.59)	
III	5	58	148				1.71(1.23–2.37)	
IV	1	18	66				1.15(0.68–1.95)	

^a^ORs were adjusted for age, sex, pack-years smoked, biomass as fuels and sample source region in a logistic regression model.

^b^*P* value from the multiple interaction test.

**Table 4:** FBAT analysis of effect of the *WWOX* SNPs on COPD risk in pedigree samples.

SNP/Haplotype	FBAT								Logistic regression model
	Allele	Frequency	fam#	S	E(S)	Var(S)	Z	*P*	OR (95%CI)
rs383362G >T	G	0.775	63	83	95.8	19.4	−2.90	0.004	1.00 (ref.)
	T	0.225	63	49	36.2	19.4	2.90	0.004	2.04(1.41–2.95)
rs10220974C >T	C	0.859	51	74	75.0	14.7	−0.261	0.794	1.00 (ref.)
	T	0.141	51	30	29.0	14.7	0.261	0.794	0.88(0.59–1.31)
rs3764340C >G	C	0.867	57	87	85.7	13.9	0.358	0.721	1.00 (ref.)
	G	0.133	57	27	28.3	13.9	−0.358	0.721	1.29(0.85–1.96)
rs12918952G >A	G	0.886	58	88	92.3	17.0	−1.04	0.299	1.00 (ref.)
	A	0.114	58	34	29.7	17.0	1.04	0.299	1.41(0.92–2.15)
rs12828G >A	G	0.762	69	113	105.2	22.1	1.67	0.096	1.00 (ref.)
	A	0.238	69	37	44.8	22.1	−1.67	0.096	0.85(0.62–1.17)
Haplotype^a^	G-C	0.706	69.0	100.0	109.0	22.3	−1.92	0.055	1.00 (ref.)
	T-C	0.148	46.2	39.0	28.7	16.3	2.55	0.011	1.64(1.11–2.41)
	G-G	0.107	40.3	16.0	19.6	9.4	−1.18	0.239	1.15(0.73–1.84)
	T-G	0.039	14.8	9.0	6.6	3.3	1.30	0.193	1.88(0.95–3.73)

Abbreviation: fam#: Number of nuclear families informative for the FBAT analysis.

S: Observed transmission for each allele; E(S) = Expected transmission for each allele.

Var(S): Variance of the observed transmission for each allele; Z score: Positive values indicate increased transmission and negative values indicate reduced transmission to affected individuals.

^a^Haplotypes generated by the rs383362G >T and rs3764340C >G polymorphisms. G-C: rs383362G-rs3764340C; T-C: rs383362T-rs3764340C; G-G: rs383362G-rs3764340G; T-G: rs383362T-rs3764340G.
