# Supplementary material for: The role of WWOX polymorphisms on COPD susceptibility and pulmonary function traits in Chinese: a case-control study and family-based analysis
Source: Sci Rep. 2016 Feb 23;6:21716. doi: 10.1038/srep21716 (PMC4763216; doi:10.1038/srep21716)
Supplement: Supplementary Table S1-2 [file srep21716-s1.doc]

**The role of WWOX polymorphisms on COPD susceptibility and pulmonary function traits in Chinese: a case-control study and family-based analysis**

Chenli Xie^1,2^, Xiaoliang Chen^1,3^, Fuman Qiu^1^, Lisha Zhang^1^, Di Wu^1^, Jiansong Chen^1^, Lei Yang^1^, Jiachun Lu^1§^

**Supplementary Table S1. Frequency distributions of demographic characteristics, possible risk factors and GOLD stages in COPD cases and controls.**

| Variables | **Southern Chinese** | | |  | **Eastern Chinese** | | |
| --- | --- | --- | --- | --- | --- | --- | --- |
|  | Cases  n (%) | Controls  n (%) | *P* ^a^ |  | Cases  n (%) | Controls  n (%) | *P* ^a^ |
| Total No. of subjects | 1025 | 1061 |  |  | 486 | 616 |  |
| Age (years) |  |  |  |  |  |  |  |
| ≤ 60 | 463(45.2) | 507(47.8) | 0.231 |  | 241(49.6) | 292(47.4) | 0.471 |
| > 60 | 562(54.8) | 554(52.2) |  |  | 245(50.4) | 324(52.6) |  |
| Sex |  |  |  |  |  |  |  |
| Male | 610(59.5) | 638(60.1) | 0.773 |  | 273(56.2) | 345(56.0) | 0.956 |
| Female | 415(40.5) | 423(39.9) |  |  | 213(43.8) | 271(44.0) |  |
| Smoking status |  |  |  |  |  |  |  |
| Ever | 499(48.7) | 424(40.0) | <0.001 |  | 215(44.2) | 227(36.8) | 0.013 |
| Never | 526(51.3) | 637(60.0) |  |  | 271(55.8) | 389(63.2) |  |
| Pack-years smoked |  |  |  |  |  |  |  |
| ≥20 | 314(30.6) | 214(20.2) | <0.001 |  | 148(30.5) | 129(20.9) | 0.025 |
| <20 | 185(18.1) | 210(19.8) |  |  | 67(15.2) | 98(15.9) |  |
| 0 | 526(51.3) | 637(60.0) |  |  | 271(55.8) | 389(63.2) |  |
| Biomass as fuels |  |  |  |  |  |  |  |
| Yes | 174(17.0) | 91(8.6) | <0.001 |  | 33(6.8) | 19(3.1) | 0.004 |
| No | 851(83.0) | 970(91.4) |  |  | 453(93.2) | 597(96.9) |  |
| Drinking status |  |  |  |  |  |  |  |
| Ever | 186(18.2) | 209(19.7) | 0.366 |  | 97(20.0) | 127(20.6) | 0.788 |
| Never | 839(81.8) | 852(80.3) |  |  | 389(80.0) | 489(79.4) |  |
| Gold stages |  |  |  |  |  |  |  |
| I | 359(35.0) |  |  |  | 213(43.8) |  |  |
| II | 356(34.7) |  |  |  | 206(43.4) |  |  |
| III | 217(21.2) |  |  |  | 54(11.1) |  |  |
| IV | 93(9.1) |  |  |  | 13(2.7) |  |  |

*^a^ P* values for a two-sided χ^2^ test.

**Supplementary Table S2.** Primary information on the TAQMAN assay of five tagSNPs in the *WWOX* gene.

| SNP, rs no. | Primers | Probes *^a^* | Color (genotype) |
| --- | --- | --- | --- |
| rs12828G>A | CAGGTGGCAAAGTAC  TTGTCATAGA (forward) | FAM-CTTTGCTAATGCTAT***G***CA | Blue (GG) |
|  | CCTTCCTGCCTTTCAA  GGTATCT (reverse) | HEX-CTTTGCTAATGCTAT***A***CA | Red (AA) |
|  |  |  | Green(AG) |
| rs12918952G>A | TTGATACCATGAACTA  CACTTGCTGTT (forward) | FAM-CCAAGGTAGAA***G***CAA | Blue (GG) |
|  | TTGAATGCTTCAGCAA  AATGCT (reverse) | HEX-CCAAGGTAGAA***A***CAA | Red (AA) |
|  |  |  | Green(AG) |
| rs10220974C>T | CAATAACTGTAGTGG  AGTGAT (forward) | FAM-CTGTGCCTC***C***TCAGGT | Blue (CC) |
|  | AGGAAGGAAAATAA  AAGAAAG (reverse) | HEX-CTGTGCCTC***T***TCAGGT | Red (TT) |
|  |  |  | Green(CT) |
| rs3764340C>G | TTCCTATTTTTAAGAT  TTACAGA (forward) | FAM-TCGCCTCTCT***C***CAACAAA | Blue (CC) |
|  | CTCGTTGGAGAAGA  GGATGTT (reverse) | HEX-TCGCCTCTCT***G***CAACAAA | Red (GG) |
|  |  |  | Green(GC) |
| rs383362G>T | ACAGATCCGCAAGA  GTAAAGGAA (forward) | FAM-TAAGAGCA***G***TCACAACAG | Blue (GG) |
|  | TTCCCATTGGTACTTA  AGATTTTTCA (reverse) | HEX-TAAGAGCA***T***TCACAACAG | Red (TT) |
|  |  |  | Green(TG) |

*^a^* Bold italic nucleotides indicate the polymorphic sites in probes.
